# Content development and validation for a mobile application designed to train family caregivers in the use of music to support care of people living with dementia

**DOI:** 10.3389/fmed.2023.1185818

**Published:** 2023-05-12

**Authors:** Zara Thompson, Jeanette Tamplin, Tanara Vieira Sousa, Romina Carrasco, Libby Flynn, Karen E. Lamb, Amit Lampit, Nicola T. Lautenschlager, Kate McMahon, Jenny Waycott, Adam P. Vogel, Robyn Woodward-Kron, Phoebe A. Stretton-Smith, Felicity A. Baker

**Affiliations:** ^1^Faculty of Fine Arts and Music, University of Melbourne, Melbourne, VIC, Australia; ^2^School of Computing and Information System, University of Melbourne, Carlton, VIC, Australia; ^3^Melbourne School of Population and Global Health, University of Melbourne, Carlton, VIC, Australia; ^4^Academic Unit for Psychiatry of Old Age, Department of Psychiatry, University of Melbourne, Parkville, VIC, Australia; ^5^NorthWestern Mental Health, Royal Melbourne Hospital, Parkville, VIC, Australia; ^6^Center for Neuroscience and Speech, University of Melbourne, Parkville, VIC, Australia; ^7^Division of Translational Genomics of Neurodegenerative Diseases, Hertie Institute for Clinical Brain Research, University of Tübingen, Germany and Center for Neurology, University Hospital Tübingen, Tübingen, Germany; ^8^Redenlab Inc., Melbourne, VIC, Australia; ^9^Department of Medical Education, Melbourne Medical School, University of Melbourne, Parkville, VIC, Australia; ^10^Norwegian Academy of Music, Oslo, Norway

**Keywords:** dementia, technology, music therapy, eHealth, caregiver

## Abstract

**Background:**

Music therapy is increasingly recognized as an effective support for people living with dementia. However, with incidences of dementia increasing, and limited availability of music therapists, there is a need for affordable and accessible ways that caregivers can learn to use music-therapy based strategies to support the people they care for. The MATCH project aims to address this by creating a mobile application that can train family caregivers in the use of music to support people living with dementia.

**Methods:**

This study details the development and validation of training material for the MATCH mobile application. Training modules developed based on existing research were assessed by 10 experienced music therapist clinician-researchers, and seven family caregivers who had previously completed personalized training in music therapy strategies via the HOMESIDE project. Participants reviewed the content and scored each training module based on content (music therapists) and face (caregivers) validity scales. Descriptive statistics were used to calculate scores on the scales, while thematic analysis was used to analyze short-answer feedback.

**Results:**

Participants scored the content as valid and relevant, however, they provided additional suggestions for improvement via short-answer feedback.

**Conclusion:**

The content developed for the MATCH application is valid and will be trailed by family caregivers and people living with dementia in a future study.

## Background

According to The World Alzheimer’s Report, there are over 50 million people living with dementia worldwide, with this figure predicted to double every 20 years (1, 2). Approximately 84% of people living with dementia reside at home and are supported by informal caregivers (usually close family members) (3). Most care provided by informal caregivers relates to activities of daily living (ADLs), which can average approximately 5 h per day (3).

Dementia symptoms relating to changes in mood, agitation, and subsequent behavior are often regarded as distressing, and can adversely impact the wellbeing of both the person with the diagnosis and their caregiver (4–6). These symptoms, often referred to as Behavioral and Psychological Symptoms of Dementia (BPSD) or neuropsychiatric symptoms, include depression, anxiety, and agitation, and can affect up to 90% of people living with dementia over the course of their illness (4, 7). Managing moderate to severe agitation and other changed behavior in people living with dementia can overwhelm informal caregivers’ capacity to cope, leading to potential for depression, burnout, and increased morbidity and mortality in caregivers (4, 8). Further, the costs of informal care can rise with increasing severity of agitation/aggression, affective changes and psychosis-related symptoms over time (9, 10). Therefore, interventions or supports are needed that can reduce and regulate these symptoms in order to improve and maintain quality of life and psychological wellbeing for people living with dementia and their caregivers, as well as reducing economic costs relating to these symptoms (10, 11). Pharmacological interventions are often employed to manage neuropsychiatric symptoms for people living with dementia, however, these can be of limited benefit as they can lead to worsening agitation and other adverse health outcomes (12, 13) or contraindications due to polypharmacy (14). There is therefore also a need for more interventions or supports that are able to address neuropsychiatric symptoms and support positive wellbeing for people living with dementia (15) as well as the wellbeing of informal caregivers (16).

### Music therapy and dementia

Music therapy programs are a promising non-pharmacological approach to address the regulation of BPSD including agitation, and support care provision and transition (17, 18) Reviews report compelling evidence that music interventions delivered by a qualified music therapist can reduce levels of depression, enhance quality of life, and promote social connectedness for people living with dementia and their informal carers (19–21). Within these programs, music therapists draw on the potential of music to orient, engage, calm, and evoke memories and emotions (22). The increasing incidence of dementia, and the small number of credentialed music therapists available to support care suggest that scalable innovative options that involve caregivers and make use of the unique power of music to support care are in urgent need of development and validation.

**Figure 1:**
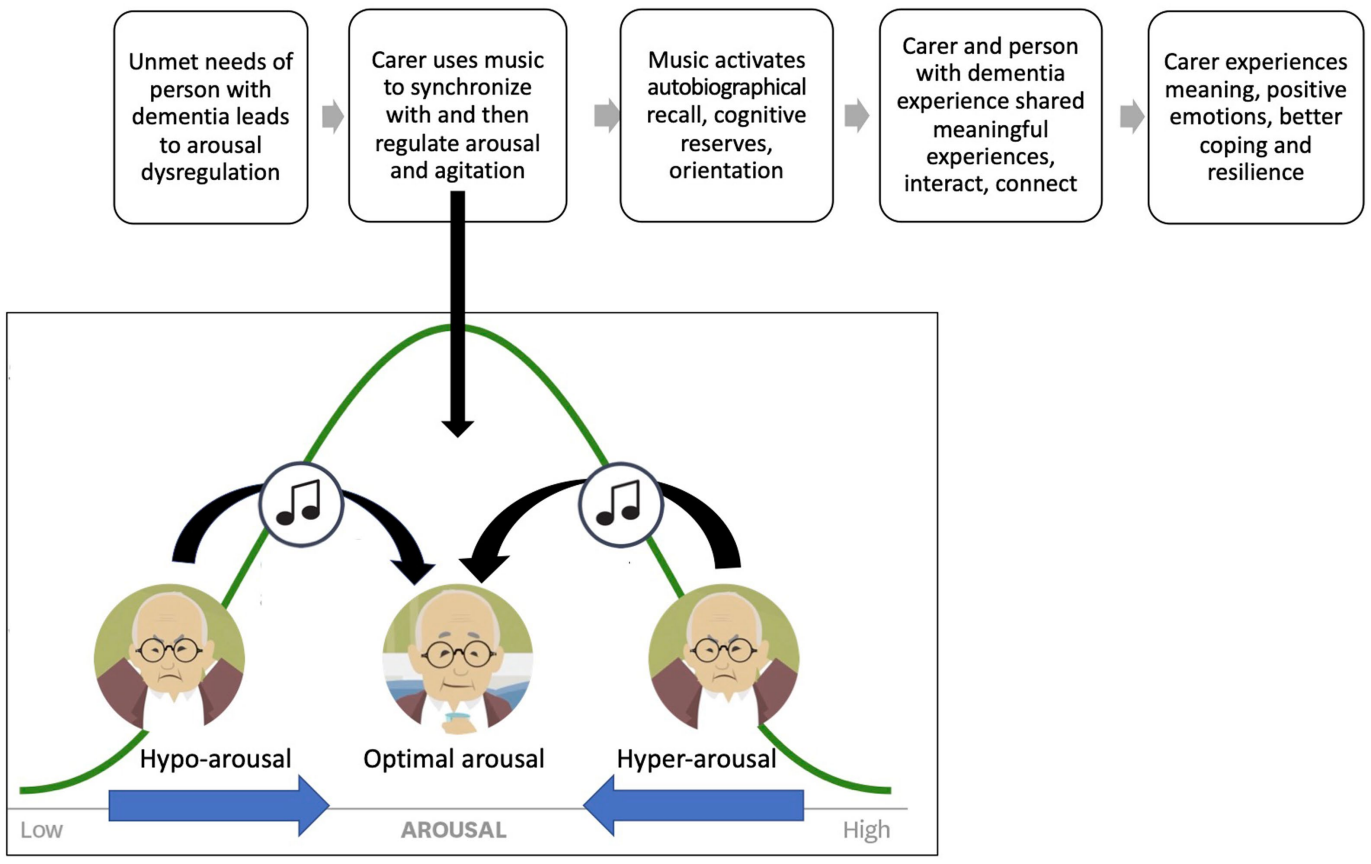
Musical attunement for arousal and agitation regulation.

HOMESIDE, a program that provides informal family caregivers with training in the intentional use of music to support care, has preliminary evidence to support its effectiveness (23), and a large trial has recently been completed (24, 25). The approach trains caregivers on principles of musical attunement to regulate arousal and agitation. Attunement is defined as sensitively and musically responding to a person’s musical and non-musical expression to tune in empathically (26) (Figure 1). However, even with this train-the-caregiver model, there are not enough music therapists to deliver this program for the vast numbers of people living with dementia worldwide.

### The MATCH app

To partially solve this workforce shortage, we have translated the content being taught by music therapists during these caregiver-training sessions into digital content for a bespoke mobile application titled Musically Attuned Technology–Care via eHealth (MATCH). This mobile application is referred to as the MATCH app. eHealth adaptations of established in-person interventions are common and offer opportunities to scale up access (27). However, the development of any eHealth intervention needs to be rooted in empirical evidence, clinical experience, and clearly defined mechanisms of change (28). A meta-analysis of eHealth interventions designed to impact changes in behavior found that interventions that linked theoretical constructs to intervention techniques had larger effect sizes (29). Our MATCH initial prototype (MATCH-P) applies the same theoretical construct of musical attunement (24) utilized in the therapist-delivered HOMESIDE music intervention (Figure 1), and trains caregivers through a mobile application to implement music-based techniques.

### Developing eHealth solutions

Kramer-Jackman and Popkess-Vawter (30) outline five steps involved in developing eHealth applications: (1) establish content; (2) establish eHealth literacy; (3) establish technology delivery; (4) establish expert usability; and (5) establish participant usability. The current study reports on the development of the MATCH app content and assessment of the validity of this content, based on an evaluation of both face and content validity (step 1).

Face validity is the process by which participants judge items on a measurement instrument to confirm whether the items proposed in the tool are congruent with the constructs and objectives of the instrument (31). However, it has also been used in eHealth development and validation to confirm that the content delivers what it is intended to (27, 32). Real life simulations embedded within eHealth applications, such as video demonstrations and case studies, can be tested to see if they are able to replicate real-life situations (30).

Content validity refers to the extent to which the components of the intervention activities are related to the underlying target construct and, therefore, most likely to be effective in achieving the intended goal (30, 33). In the case of MATCH, this relates to the extent to which the proposed learning objectives of the digitally delivered training content connect with relevant music therapy theories. Assessments test the relevance, likely effectiveness, representativeness of the content domain, accuracy, and clarity of the content. In our study, we aimed to evaluate whether the series of digital training modules met face and content validity with respect to our learning objectives, and in comparison with the HOMESIDE therapist-delivered training program. Specifically, we aimed to determine whether:

1. the modules within the MATCH prototype reflect the HOMESIDE training and whether they are engaging, clear, and relatable to people with lived experience of caring for someone with dementia (face validity);
2. the training modules adequately describe concepts of music-stimulated reminiscence, music attunement and music for care (content validity);
3. the training modules adequately provide instructions for safe and effective use of music for movement, music for relaxation, reminiscence, care, and music attunement (content validity);
4. the content is sufficiently comprehensive (no omissions, over-explanations, and irrelevant information; content validity);
5. the learning objectives of the training are clear to the user (content validity); and
6. the design of the modules is engaging, including the right mix of video, and textual information (content validity).

## Methods

### Development of content

The online training content was developed following several iterations of the original protocol published by Baker et al. (23). This caregiver training program, originally titled “Meaningful Musical Moments,” was informed by clinical experience and music therapy research, and comprised three modules: song singing, gentle movement to music, and listening to quiet, relaxing music. This program was later revised and expanded by the HOMESIDE research team, informed by consumer perspectives, clinical experience, and recent research (25). The revised HOMESIDE music intervention included four primary activities: (1) singing familiar songs with facilitated meaningful discussion; (2) movement to music; (3) music for relaxation; and (4) playing musical instruments using existing or homemade musical instruments (24, 25).

To address the need for a scalable approach, the team embarked on developing a program that could be delivered via a digital app. We subsequently developed specific learning objectives and additional learning modules, incorporating further input from people with lived experience of dementia or caring for a person with dementia, who were part of the HOMESIDE Public and Patient Involvement committee.

**Figure 2:**
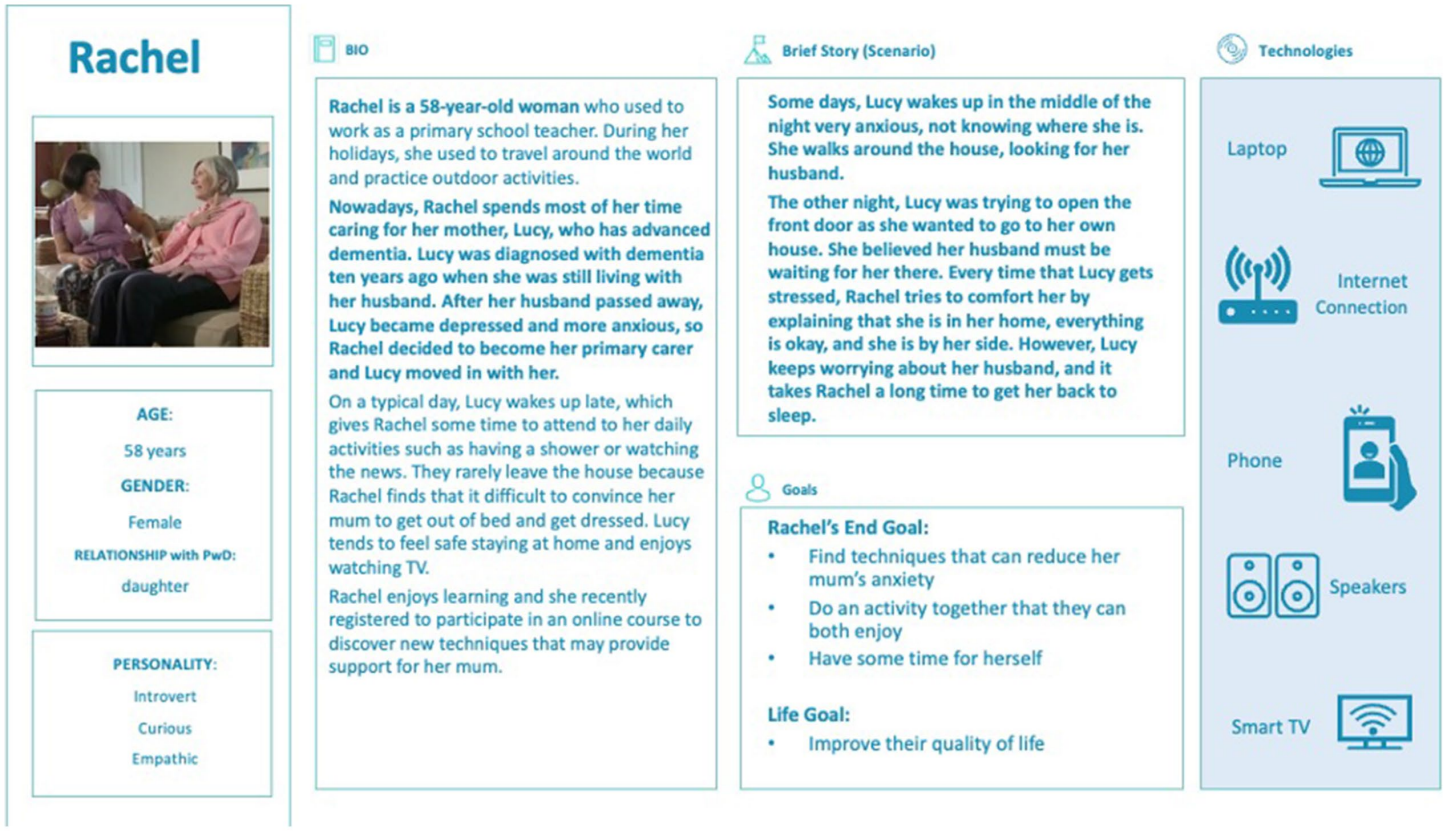
Example of a persona developed for MATCH, based on HOMESIDE data.

Personas were developed that represented the typical characteristics of the caregivers enrolled in the HOMESIDE study (34). Personas are fictional but realistic characters created based upon the synthesis of different participant-character types, which help researchers and designers understand participants’ or users’ needs, experiences, behaviors, and aspirations (35). Personas were developed by reviewing demographic information, participant diary entries, and transcripts of participant interviews. An example of a persona is detailed in Figure 2. From these personas, scripted demonstrations of music intervention implementation were constructed and organized into training modules.

The final set of content comprised eight training modules, with each including a series of scripted videos, case studies modeling the use of music-based strategies, a problem-solving tab (listing scenarios of unanticipated reactions and suggested responses), and safety checklists. The modules included information on how to use the MATCH app, the science behind the therapeutic effects of music, music for relaxation, music attunement, music for reminiscence, music to support personal care, and movement to music.

### Recruitment and participants

Ethics approval for this study was granted by The University of Melbourne Human Ethics Committee (ethics ID: 2280). People who are familiar or unfamiliar with the topic can help evaluate clarity, comprehensiveness of content, engagement, and understanding (30, 31). Informed by previous research (33, 36, 37), we aimed to recruit 10 expert music therapists and 10 people with lived experience of caring for a person with dementia (expert caregivers) via purposive sampling. Expert caregiver participants (CGs) were randomly selected from a pool of 30 participants who had completed the HOMESIDE intervention at the time of recruitment for this study. CGs were contacted via email with a plain language statement and consent form and were offered compensation in the form of a gift voucher for their participation.

Expert music therapist participants (MTs) were randomly selected from a pool of music therapy researchers with at least 5 years clinical experience and who had published at least two peer-reviewed articles on music therapy with people living with dementia and/or caregivers of people living with dementia (38). A list of potential expert MT participants was generated through database searches of published music therapy studies of people living with dementia, noting the primary author, commencing with the most recent publications (2021) and working backward until we had reached a total of 30 experts. We randomly selected 10 MTs from the total pool, and continued to do so until we had obtained 10 consenting participants. The selected MTs were contacted via email with a brief invitation to participate and a link to the plain language statement and online consent form. Expert MT participants were also offered compensation in the form of a gift voucher for their participation.

### Data collection

#### Face validity

Consenting participants were provided with links to review the content of each module via an online survey. Study data were collected and managed using REDCap (Research Electronic Data Capture) tools hosted at The University of Melbourne (39, 40). REDCap is a secure, web-based software platform designed to support data capture for research studies, providing: (1) an intuitive interface for validated data capture; (2) audit trails for tracking data manipulation and export procedures; (3) automated export procedures for seamless data downloads to common statistical packages; and (4) procedures for data integration and interoperability with external sources.

Consenting CGs were asked to review the introductory modules (modules 1–3) via short-answer responses only, as these modules outlined music therapy principles and science behind the music that were not features of their HOMESIDE training. CGs were then asked to score content in the remaining modules (modules 4–8) using the Face Validity scale. Specifically, we asked CGs to:

1. Rate how the introductory videos compared to their therapist-delivered training that they received during HOMSIDE.
2. Rate case examples to determine whether they were (a) engaging to view; (b) understandable in terms of language use and clarity of presentation; and (c) relatable to people with lived experience (41).
3. Rate the written material of each module to determine whether they found it useful in relation to their own experiences of caregiving.

Each criterion within the modules reviewed was rated using a four-point Likert scale (strongly disagree = 1, disagree = 2, agree = 3, and strongly agree = 4). CGs were also asked to provide general feedback for each module and item via short-answer response.

Caregiver participants were randomly assigned the order of modules 4–8 to review; this was adopted to ensure that all modules were reviewed should any participants withdraw prior to completing all modules. All responses were entered directly into a REDCap database (39, 40).

#### Content validity

Consenting MTs were provided with links to review the content of each module via an online survey. All MTs reviewed the introductory modules (modules 1–3) and were randomly assigned other remaining modules (modules 4–8). This process ensured that all modules were reviewed should any participants withdraw prior to reviewing all modules. MTs were asked to score the content for each module via an online survey version of the Content Validity Survey Tool, adapted from the Suitability Assessment of Materials tool (42). MTs rated each module according to how accurately the learning objectives were represented in the content, how comprehensive the content was, and how clear it was, using a four-point Likert Scale (1 = strongly disagree to 4 = strongly agree). MTs were reminded that this content had to be digestible and understandable to a lay person. They were also invited to provide general feedback for each module via short answer responses. All responses were collected via a REDCap online survey form (39, 40).

### Analysis

#### Face validity

All items scoring 3 or 4 (agree or strongly agree) were considered face valid for those criteria, and those scoring 1 or 2 (strongly disagree or disagree), were considered face invalid. Each module needed to have at least four of the five criteria rated as face valid by at least 80% of CG participants to be retained (43).

#### Content validity

All items were scored using a calculation of an adapted content validity index (CVI) (44). The CVI is typically used to measure relevance, clarity, and necessity on individual items (I-CVI) and for the whole scale (S-CVI). While the S-CVI/Universal agreement ≥0.8 and a S-CVI/Average ≥ 0.9 have both been found to have excellent content validity (45, 46), we chose to use the more conservative Universal agreement (S-CVI/UA). Based on previous examples (44, 46), we chose to rate each learning objective (I-CVI) according to three criteria: *accuracy* of the content, *comprehensiveness* of the content, and *clarity* of the content using a four-point Likert scale from (1 = strongly disagree to 4 = strongly agree) (47). All items scoring 3 or 4 were considered content valid, and those scoring 1 or 2, content invalid (43). The proportion of MT experts who rated the content as valid for each criterion within each learning objective was calculated by dividing the number of experts who rated that content as valid by the total number of experts. If the proportion of raters for I-CVI was >0.79 for a criterion on an item, the content was considered valid. If the proportion of raters rating the item as valid scored between 0.70–0.79, then the item would be categorized as requiring revision. If a value was below 0.7, the content would be categorized as requiring major revision or removal from the content (46). To calculate the content validity of the whole MATCH app content (S-CVI), the proportion of valid criteria was calculated by summing the number of criteria scored as valid (that is, the number of I-CVI items that obtained a score of >0.7) and dividing this by the total number of criteria assessed.

#### Short answer responses

Short answer responses were grouped by participant type (CG or MT) and module, producing 12 data groups. Data were then analyzed using a six-step deductive Thematic Analysis method (48) via MAXQDA software (49). Data were coded by author 1 and confirmed by the last author according to three pre-specified overarching themes (step 1): (i) Content is Appropriate and Well Received; (ii) Content that Requires Changing/Adapting; and (iii) Design/Esthetic Aspects that Require Improvement. Data for the first data group were read for familiarity (step 2), and then coded under one of the three overarching themes (step 3). Similarly coded data under each theme were grouped and subthemes were created (step 4). This process was repeated for each data group, until all groups were completed (step 5). Data from the CG and MT groups were compared and grouped to provide a final set of subthemes (step 6). Once the analysis was completed, we used these findings to identify potentially problematic or redundant content and to refine the remaining intervention content as needed (44).

## Results

Between January and April 2022, we invited 18 MTs and 10 CGs to participate; of these, 11 MTs and nine CGs consented to participate in the study. One CG withdrew before commencing, while two CGs and one MT only partially completed the study. Both participant groups rated the content and face validity of items as high overall. MTs assessed *overall* content validity as 100% for each domain. When assessing each module, high scores were ascribed for accuracy, comprehensiveness, and clarity across most items (Table 1).

**Table 1:** Content validity assessed by expert music therapists.

	Accuracy	Comprehensiveness	Clarity
Total scores for MATCH music training program content (S-CVI)	1.000	1.000	1.000
Module 1: introduction to MATCH App (I-CVI; *n* = 11)			
Item 1.1: responses to music	0.909	1.000	0.909
Item 1.2: impact on mood	1.000	1.000	0.818
Item 1. 3: when to implement	1.000	1.000	1.000
Module 2: the science behind the music (I-CVI; *n* = 11)			
Item 2.1: music and memory	1.000	0.909	0.909
Item 2.2: memory and emotions	0.909	0.909	1.000
Item 2.3: engagement with music	1.000	1.000	1.000
Module 4: music for relaxation (I-CVI; *n* = 10)			
Item 4.1: contexts for relaxation	1.000	1.000	1.000
Item 4.2: musical characteristics	1.000	1.000	1.000
Item 4.3: assessing suitability	1.000	1.000	1.000
Item 4.4: environmental awareness	1.000	1.000	1.000
Item 4.5: guided relaxation	1.000	0.900	1.000
Item 4.6: music and imagery	1.000	0.900	1.000
Module 5: music attunement (I-CVI) (*n* = 10)			
Item 5.1: identifying energy levels	1.000	1.000	1.000
Item 5.2: selecting appropriate music	1.000	1.000	1.000
Item 5.3: adapting music	0.900	1.000	1.000
Item 5.4: applying attunement	1.000	1.000	1.000
Module 6: music for reminiscence (I-CVI; *n* = 10)			
Item 6.1: selecting appropriate music	1.000	1.000	1.000
Item 6.2: recognizing non-verbal responses to music	1.000	1.000	0.900
Item 6.3: identifying and responding to distress	0.800	0.900	0.900
Item 6.4: initiating conversation	1.000	1.000	1.000
Item 6.5: managing conversation	0.900	0.900	0.900
Item 6.6: repetition	0.900	0.900	0.900
Item 6.7: including other media	1.000	1.000	1.000
Module 7: music to support personal care (I-CVI; *n* = 10)			
Item 7.1: selecting appropriate music	1.000	1.000	1.000
Item 7.2: improvisation skills	1.000	1.000	1.000
Module 8: movement to music (I-CVI; *n* = 11)			
Item 8.1: recognizing escalation	1.000	0.818	1.000
Item 8.2: initiating music response	0.909	1.000	0.909
Item 8.3: selecting appropriate music	0.909	0.818	0.909
Item 8.4: environmental safety	1.000	1.000	0.909
Item 8.5: positioning	1.000	1.000	0.909
Item 8.6: engagement strategies	1.000	1.000	1.000
Item 8.7: suitability of exercises	0.909	0.909	0.909
Item 8.8: recognizing negative responses	0.909	0.818	0.909
Item 8.9: adapting music to maximize engagement	1.000	0.909	1.000

S-CVI, scale–content validity index; I-CVI, item–content validity index.

*Overall* Face Validity was also scored highly by CGs (Table 2). Two items (Items 3c and 4 in Module 4) scored lower than 80%; however, as the overall score for the module was over 80%, the module was deemed face valid and retained.

**Table 2:** Overall face validity—assessed by expert caregivers.

	Comparability to HOMESIDE	Engaging	Understandable	Realistic	Usefulness
Overall face validity of training modules	0.970	0.980	0.980	0.950	0.870
Module 4: music for relaxation (*n* = 8)					
Item 4.1: instructional video	1.000				
Item 4.2: case study 1		1.000	1.000	1.000	
Item 4.3: case study 2		0.875	0.875	0.750	
Item 4.4: written content					0.625
Module 5: music attunement (*n* = 8)					
Item 5.1: instructional video	0.875				
Item 5.2: case study 1		1.000	1.000	1.000	
Item 5.3: case study 2		1.00	1.000	0.875	
Item 5.4: written content					1.000
Module 6: music for reminiscence (*n* = 6)					
Item 6.1: instructional video	1.000				
Item 6.2: case study 1		1.000	1.000	1.000	
Item 6.3: case study 2		1.000	1.000	0.875	
Item 6.4: written content					1.000
Module 7: music to support personal care (*n* = 6)					
Item 7.1: instructional video	1.000				
Item 7.2: case study 1		1.000	1.000	1.000	
Item 7.3: written content					1.000
Module 8: movement to music (*n* = 8)					
Item 8.1: instructional video	1.000				
Item 8.2: case study 1		1.000	1.000	1.000	
Item 8.3: case study 2		1.000	1.000	1.000	
Item 8.4: case study 3		1.000	1.000	1.000	
Item 8.5: written content					0.875

### Qualitative results

Analysis of the short answer responses revealed nuanced feedback relating to the three predefined themes: (1) Content is appropriate and well received; (2) Content that requires changing/adapting; and (3) Design/Esthetic aspects that require improvement. These themes and subthemes are reported below.

#### Theme 1: content is appropriate and well received

Responses to open ended questions from CGs and MTs indicated that the training content designed for the app was well received and appropriate. In particular, the CGs provided nuanced responses, which were synthesized into five subthemes.

##### Content is accessible

Caregiver participants felt that the way that the content was delivered was accessible and easy to understand. CGs felt that the videos were “*easily relatable for lay people, family and caregivers alik*e” and that the “*videos were of perfect length and demonstrated the scenarios effectively*” (CG05).

##### Benefits to caregivers

Caregiver participants commented on how they could imagine using the app to support their daily care routines. CGs reported that the idea of providing “suggested playlists” targeting specific care needs was helpful as they sometimes did not know what to select when engaging the person with dementia. For example, one CG explained: “*some examples of suitable classical music for relaxation will be appreciated as I find it difficult to find classical music with consistent slow tempo without sudden surprises*” (CG08). Another CG felt that the proposed activities could effectively integrate music into their already busy routines:

“*Integrating music into the day and not being seen as a task is a strong point*. *Some would see it as ' one more thing' caregivers need to do*. *After walking in from an 8 or 12 hour ICU shift and [then] having to continue having my ‘care factor’ turned on, perhaps just playing some music in the background will be beneficial*” (CG05).

##### More comprehensive than HOMESIDE training

Some CGs commented that they found aspects of the MATCH content to be more comprehensive than the training that they received during the HOMESIDE study. Some participants commented that they *“learned new methods and techniques”* (CG08) and that “*there was more content in these modules than I recall with our [HOMESIDE] Zoom sessions*” (CG03).

##### Content is realistic and helpful

Caregiver participants reported that the content was reflective of their own situations and that the content was “*realistic and helpful…case study is perfect—absolutely identify with it!*” (CG10). However, several participants noted across several modules, that the case examples were not reflective of their current experiences of caring for their family member with dementia. This was most prominent in the responses relating to the “Relaxation” module, which may explain the lower score for this module (item 3, case study 2). One CG noted that the focus on agitation and re-directing in the “Attunement” module, was not relevant to them “*at this stage, however the tips are useful for future reference”* (CG03). This highlights the need to reinforce that some content may have more relevance for use during different stages of the disease progression.

#### Theme 2: enhancing existing content

While CGs and MTs each scored the existing content highly, and felt that no major changes were essential, they also provided detailed feedback on ways that the content could be further enhanced. Three subthemes were developed based on where feedback from both CGs and MTs converged (*Diversify Examples; Additional Uses for Attunement; and Module on Caregiver Needs*). Two additional subthemes relate only to feedback from CGs (*Ensuring Consent/Person-Centered Care*) and MTs (*Simplifying Language*).

##### Diversify examples

Caregiver participants and MTs acknowledged that the current case examples lacked diversity in age, gender, relationship, diagnosis, stage/symptoms, and cultural background. “*I wondered whether diversity has been considered in the case studies, particularly in terms of ethnicity, but perhaps also in terms of other caring relationships,* e.g.*, same sex couples*.” (MT03). One CG highlighted that people with multiple conditions may need additional strategies: “*[The person in the case example] only has dementia—if patient has other conditions, it is not so easy!”* (CG07).

Some CGs perceived that the case examples only illustrated positive responses to music, neglecting to demonstrate examples of negative responses and what to do when these occurred. One CG explained how several of the training videos “*make it all look easier than it actually is*” (CG10). This sentiment was echoed by MTs, who felt that “*it would be worth mentioning that sometimes [strategies] might not work, and it’s not because the caregiver should have done anything differently—the same approach might produce the desired effect next time*” (MT01).

One CG noted that having examples of how to include other family members would be helpful in future iterations of the MATCH app:

*“It would also be good if there are younger kids around or another person to make it a group activity*. *Sometimes if kids are involved it also helped”* (CG07)

##### Additional uses for attunement

Caregiver participants and MTs reported that the attunement module would benefit from additional examples of how to use attunement to impact mood-states other than agitation. MTs made suggestions for additional ways that attunement could be exemplified, for example:

“*Maybe having one or two more examples of different energy levels would be useful—for example, high energy level but without distress/agitation, or low energy level but not relaxed but sad/preoccupied/indifferent*.” (MT06)

##### Module on caregiver needs

Music therapist participants highlighted a lack of content related to supporting caregivers directly. The relaxation module was highlighted as a space that could include specific strategies for caregiver self-care. Another MT commented that they “*wondered if it’s possible to acknowledge how much the caregiver might need this relaxation and perhaps suggest they could do this by themselves without the person when they have a chance, to self-care*” (MT15). This sentiment was echoed by CGs: “*I can relate as well as a caregiver*. *I put music on for my own relaxation*” (CG06).

##### Ensuring consent/person-centered care

Some CGs observed that as each of the case examples presented depict a “successful” session, there was a lack of content demonstrating how to respect a person with dementia’s autonomy to reject an activity. One CG noted that care recipients *“may not like lyrics”* that are created by caregivers, and that *“some activities are not relevant”* depending on circumstances (CG07). Another CG highlighted the importance of following the lead of the care recipient:

*“I wouldn't press on with my agenda for ‘relaxation’ if he wasn't ready—I think it works best to let him set the pace—do what he wants to do—then try again in say half an hour…”* (CG10).

##### Simplifying language

Several MT participants commented that they felt some of the language used in the training videos could be simplified and de-jargonised:

*“Some words in the introduction may be too complicated: noticed 'sedative', 'cognition', 'tempo', 'evoke'*. *Just wondering if simpler words might be helpful?”* (MT15)

This was highlighted as especially important for caregivers where English is not their first language:

*“I was just wondering whether the term 'imagery' is a commonly understood term in English or whether it should be accompanied by a very brief description when first mentioned*.*”* (MT06)

Music therapist participants also suggested more clarity regarding the terminology used to describe emotions, suggesting that the language used may oversimplify how care recipients may experience different emotions while listening to music:

*“Many relevant tips are given concerning different types of distressful behaviors*. *However, I think it would be beneficial to address more clearly, that sometimes crying is a good thing to share, and not necessarily a response where the caregiver immediately should stop the song and change activity*.*”* (MT04)

#### Theme 3: improving esthetics and design

Music therapist and CG participants highlighted concerns regarding the quality of some of the training videos, as well as potential improvements in how accessibly the information was presented.

##### Length of training

Participants commented that some videos were quite long, and could be repetitive at times. One CG felt that there was “…*some redundancy in the first and second module with repeated information”* (CG03). One MT raised that signposting the length of videos would help with accessibility:

*“I think if I was the caregiver, I would like to know approximately how long each video is, how long I should spend on it*.*”* (MT01)

##### Clarity of video recordings

Some participants noticed that the way the videos were recorded reduced the accessibility of the information being presented. One CG found that a video that featured a mirror was “*distracting”* due to reflections (CG03). Several MTs commented that, at times, they found the background music to be “…*too strong and loud*…,” which at times made it “…*hard to hear the narrator…*” (MT04). MTs also suggested that videos should have a “*text overlay…to help [anchor]…attention and understanding during the video*” (MT04). Another MT also noted that some of the visuals used were too complicated:

“I welcomed the visual aide—however, there were too many labels on the brain, and the narrative did not highlight exactly what the visual aide did. I would recommend simple images of the brain highlighting areas or pathways being discussed as it is viewed.” (MT10)

##### Clarity of written information

Music therapist participants also commented on the format of the written information (summary of the video content and suggestions for what to do when things go wrong); they felt that the information was too lengthy:

“Some texts (e.g. for Tips) are pretty long—perhaps you can consider clearer signposting e.g. bold font for key points, use of bullet points” (MT17).

## Discussion

Results of our study indicate that all of the MATCH online training modules met minimum face and content validity requirements to be retained in their current form. That said, qualitative feedback still enabled our participants to share suggestions for improving the training content and presentation, including suggestions for additional information to be included. Former HOMESIDE participants (our expert CG raters) reported that the modules were comparable with the training they received in HOMESIDE, and that the content was engaging, understandable, realistic, and useful for their contexts. Further, some commented that they experienced learning additional content from this training that they had not learned in their HOMESIDE training. There may be a few explanations for this: firstly, the HOMESIDE training was delivered live and tailored to participants’ needs (24, 25), while the expert CGs in the present study reviewed the entire training content (even modules that were not relevant to their current situation). Therefore, it is possible that the CGs reviewed content that was not presented to them during the HOMESIDE training. Secondly, the MATCH training content is arranged by presenting needs/outcomes, whereas HOMESIDE training was arranged by intervention method, with the intention of the person with dementia being present during the training (24, 25). It is possible that the different organization of content, framing of the science behind the music and intervention strategies, and targeting of CG only may have made the content easier for CGs to absorb. Finally, while the content for the MATCH training in the current version was based on the HOMESIDE protocol, additional iterations, including the integration of consumer input, may have led to a stronger, more comprehensive program.

This feedback suggests that delivering this training digitally was comparable to the in-person delivery.

The findings highlight that complex concepts, such as music-stimulated reminiscence, music attunement and music for care, were adequately conveyed to caregivers. However, qualitative feedback from MT-participants suggests there is a need to simplify the terminology used so it is understandable to a broader audience. Notably, this issue was not raised by CG-participants, although it is possible that this may be due to selection bias (participants who have an interest in participating in research may have been familiar with scientific terminology), or because CGs had some familiarity with music and dementia terminology from their experience in the HOMESIDE study. Furthermore, research indicates that use of jargon or specialized terminology can be a barrier for patient or informal caregiver education and decision making (50). Culturally responsive communication has also been found to be crucial in reducing barriers for people accessing healthcare support (51). Therefore, we feel it necessary to acknowledge the feedback from the expert MTs and further refine the language used in the modules so that it is more accessible for people who may be less familiar with the terminology, especially those for whom English is an additional language or those who have a low level of literacy. This feedback calls for the research team to invest considerable time with end users to ensure the terminology selected is well recognized, comprehensible, and has little likelihood of being misunderstood (51).

The only module to receive a face validity rating from expert CGs below 80% (“excellent”) was the relaxation module, which scored 75 and 62%, respectively, for “realistic” and “useful” domains. Although our initial method called for modules with scores below 80% to be re-evaluated and below 70% to be removed, the qualitative feedback highlighted that the reason for these scores was not because CGs felt that the module was unnecessary, but that it did not reflect their current personal circumstances. CGs commented that they felt they could see how these modules would be helpful for others, or even for themselves further down the track. This reflects previous research that found that informal caregivers of people with dementia find value in learning from others in similar circumstances so that they can prepare for what is to come (52). Therefore, we feel that no revision is required at this stage, as CGs continued to see value in the module even though it did not relate to their specific circumstance.

No concerns were raised by expert MT-participants with respect to the comprehensiveness, clarity, and accuracy of the instructional content. In particular, no concerns were raised about safety. As we are developing a “medical device,” it is critical that our training does not pose any risk (53), and safety is an aspect of app design that the research team place high priority on. Including a needs assessment, and a physical safety assessment within the app helps caregivers to identify which modules are most relevant (and safest) for their use. For the movement to music module, we have consulted with a physiotherapist and placed a disclaimer on all instructional videos, as well as providing regular cautionary statements about safety throughout that module. Each module provides a section on “what to do in scenarios where the person being cared for does not respond in the ways intended or anticipated” in written form. Such content was deemed clear and useful by participants via the content and face validity scores. However, several participants (both MT and CG) suggested further video case examples depicting scenarios where people living with dementia did not respond as expected or responded negatively to suggested interventions. CG-participants particularly requested examples relevant to their own experience, notably related to highlighting how to respect the person with dementia’s autonomy to decline an intervention. The HOMESIDE music interventions were designed using principles of person-centered care and validation (24, 54), which have long been established as important factors of maintaining psychological wellbeing and quality of life of people living with dementia (55). The feedback from participants highlights the importance of providing multiple examples to demonstrate how person-centered care can be enacted in various scenarios, and to compensate for the lack of personalized advice that the app offers.

### Strengths and limitations

A strength of this study is that we were able to recruit a range of expert caregivers and music therapists, who provided important insights based on their expertise and lived experience. However, there is a limitation that both MTs and CGs were aware of who the research team were due to their association with the HOMESIDE study, as the lead researcher (FB) is a prominent presenter in the training videos. This may have caused participants to respond favorably. Although we were initially concerned that the high scores on the face and content validity scales might indicate a response bias, we feel that the inclusion of qualitative data has helped to provide depth and nuance to the feedback and demonstrate that participants were not simply selecting high scores to appease the researchers.

An important limitation of this study is the lack of diversity among participants, particularly expert CGs. We did not collect demographic data for this particular study; therefore, we cannot report on the diversity of participants. However, it should be noted that all expert CG participants were Australian residents and were fluent in English. While expert MTs from a range of countries were included, as we did not collect demographic data, we cannot report on the diversity of these participants either. As one subtheme for Enhancing Existing Content related to the lack of diversity (cultural, linguistic, gender, sexuality, and dementia type/stage), collecting this information is essential in future research to ensure that diverse perspectives are captured and that future iterations of the MATCH app are informed by and accessible to people with diverse backgrounds and lived experience.

### Implications for future app development and research

Changes in mood and behavior associated with dementia are often experienced as distressing, and can adversely impact the wellbeing of both the person with the diagnosis and their caregiver (4–6). Further, managing agitation and other neuropsychiatric symptoms can overwhelm a family caregiver’s capacity to cope. There is a need for interventions and supports that can reduce and regulate these symptoms in order to improve and maintain quality of life and psychological wellbeing for both the person with dementia and their caregivers (15, 16).This preliminary study suggests that the training modules developed for the MATCH app are acceptable as a way of conveying music therapy principles to support caregivers of people living with dementia. While several studies have found music interventions to be a suitable alternative to (potentially harmful) pharmacological approaches, further research is required to test whether the app can deliver the training in a format that caregivers can understand, relate to and use in their daily lives. The MATCH team plans to implement the training via the MATCH app in pilot studies and will compare the effectiveness of this format compared to the personalized delivery that HOMESIDE offers.

## Data availability statement

The datasets presented in this article are not readily available because as this data relate to review of confidential content, the data are not publicly available. Requests to access the datasets should be directed to ZT, zara.thompson@unimelb.edu.au.

## Ethics statement

The studies involving human participants were reviewed and approved by The University of Melbourne Human Ethics Committee (ethics ID: 2280). The patients/participants provided their written informed consent to participate in this study.

## Author contributions

FB initiated and led the study. FB, TV, KL, AL, NL, JW, AV, and JT obtained funding. FB, JT, LF, ZT, PS-S, KM, RC, RW-K, and JW developed the module content. ZT and TV recruited participants and monitored the data collection. ZT and FB were responsible for qualitative data analysis and ZT and TV for quantitative analysis. ZT, JT, TV, RC, LF, KL, AL, NL, KM, JW, AV, RW-K, PS-S, and FB had access to the data and contributed to interpreting the data, drafting the manuscript, and approving the final version of the manuscript and had final responsibility for the decision to submit for publication. All authors contributed to the article and approved the submitted version.

## Funding

This project was commissioned by the World Health Organization’s Arts and Health Initiative. Funding for this project was provided through a grant from the National Health and Medical Research Center—Medical Research Future Fund, Grant Number MRFF2007411.

## Conflict of interest

AV was employed by Redenlab Inc.

The remaining authors declare that the research was conducted in the absence of any commercial or financial relationships that could be construed as a potential conflict of interest.

## Publisher’s note

All claims expressed in this article are solely those of the authors and do not necessarily represent those of their affiliated organizations, or those of the publisher, the editors and the reviewers. Any product that may be evaluated in this article, or claim that may be made by its manufacturer, is not guaranteed or endorsed by the publisher.
